# Tracheal metastasis from primary oral malignant melanoma: A case report

**DOI:** 10.1097/MD.0000000000045897

**Published:** 2025-11-07

**Authors:** Yun Liu, Wenwen Guo, Shanshan Zhang, Cuixia Bian

**Affiliations:** a Clinical Medical College, Jining Medical University, Jining, Shandong, PR China; b Painless Endoscopy Nursing Department, Jining No. 1 People's Hospital, Jining, Shandong, PR China; c Department of Pulmonary and Critical Care Medicine, Jining No. 1 People's Hospital, Jining, Shandong, PR China.

**Keywords:** high-frequency electrocautery snare resection, histopathology, immunohistochemistry, oral malignant melanoma, tracheal metastasis

## Abstract

**Rationale:**

Oral malignant melanoma (OMM) is a rare but highly aggressive malignancy that frequently metastasizes to the lymph nodes, lungs, liver, brain, and bones; however, tracheal metastasis is uncommon. Such cases are rarely reported.

**Patient concerns:**

A 58-year-old man with a history of primary oral melanoma treated with surgery followed by radiotherapy and chemotherapy 2 years prior presented with respiratory symptoms. Chest computed tomography and bronchoscopy revealed a pigmented neoplasm obstructing the main trachea of the patient. Histopathological and immunohistochemical analyses of the neoplasm confirmed the presence of metastatic melanoma.

**Diagnoses:**

Tracheal metastasis of OMM.

**Interventions:**

Complete resection of the lesion within the tracheal lumen was achieved using high-frequency electrocautery snare resection via bronchoscopy.

**Outcomes:**

The patient’s airway was reestablished, and the symptoms of respiratory distress were alleviated.

**Lessons:**

Clinicians should be alert to the possibility of tracheal metastasis in patients with OMM presenting with nonspecific respiratory manifestations.

## 1. Introduction

Oral malignant melanoma (OMM) is a rare and aggressive malignancy, accounting for approximately 0.5% of all oral cancers, with the hard palate and maxillary gingiva being the predominant sites of involvement.^[1,2]^ Compared to cutaneous melanoma, mucosal melanoma exhibits more aggressive biological behavior with a predilection for locoregional recurrence and distant metastasis, consequently leading to elevated mortality rates.^[3]^ While OMM commonly metastasizes to the lymph nodes, lungs, liver, brain, and bones, tracheal involvement is exceedingly rare.^[4]^ We report a case of tracheal metastasis originating from primary OMM, focusing on its clinical characteristics and associated diagnostic and therapeutic challenges.

## 2. Case presentation

**Figure 1.**
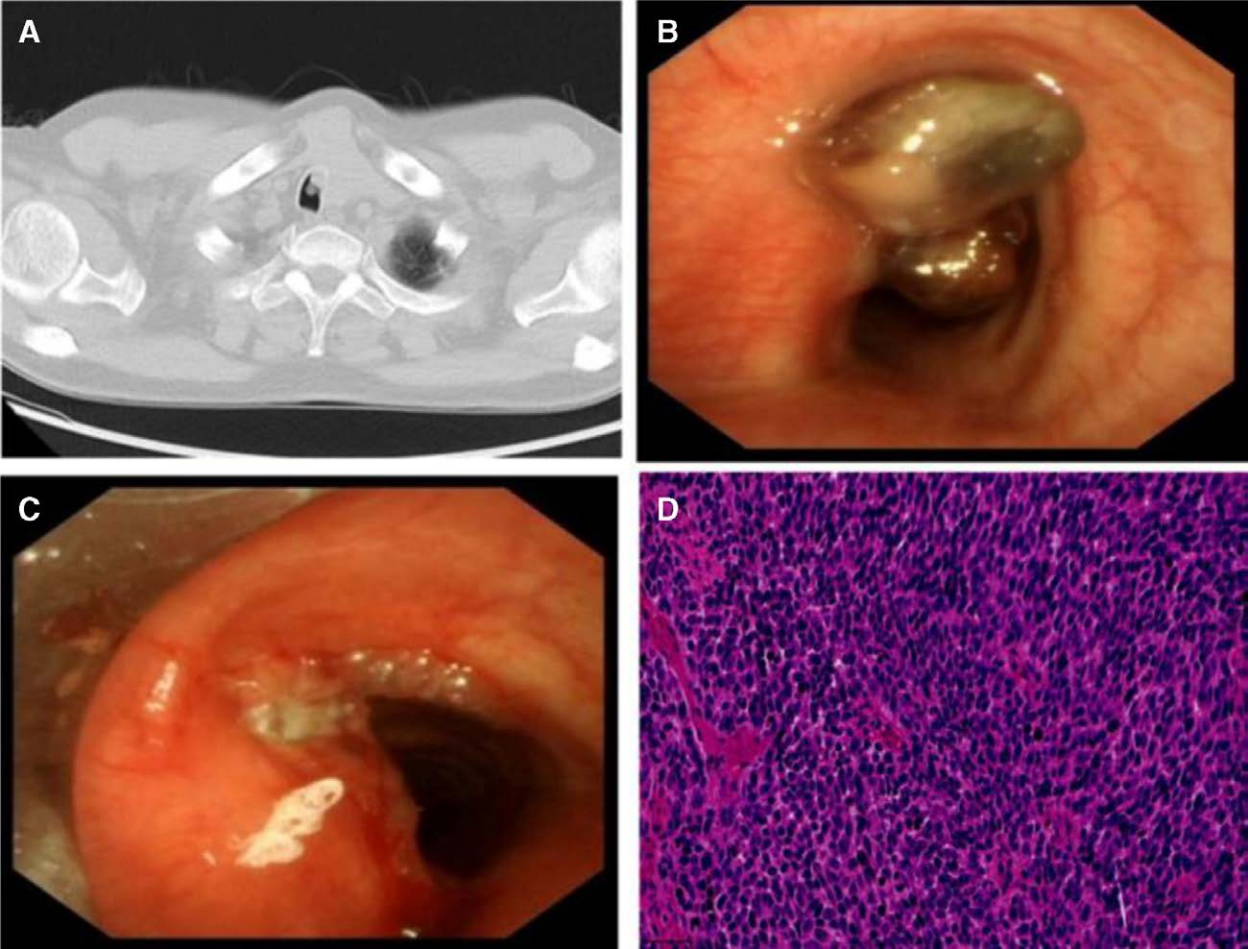
(A) CT revealing an endobronchial soft tissue density. (B) Bronchoscopy reveals a pigmented neoplasm in the main bronchus, causing partial airway obstruction. (C) Lesion is completely resected using high-frequency electrocoagulation, and airway patency is restored. (D) Histopathological examination reveals the presence of melanoma cells. CT = computed tomography.

A 58-year-old man presented to our hospital with a 1-month history of cough, expectoration, and dyspnea. Two years prior, melanoma was detected via palatal biopsy, and the patient underwent surgical excision, followed by radiotherapy and chemotherapy. Computed tomography at the time of presentation revealed tracheal soft tissue density (Fig. 1A). Subsequent bronchoscopy revealed a pigmented neoplasm in the trachea, causing approximately 70% airway obstruction (Fig. 1B). Following complete resection of the intratracheal lesion via high-frequency electrocautery snare resection, the patient’s airway was restored to patency, and symptoms of respiratory distress were alleviated (Fig. 1C). Histopathology revealed the presence of melanoma cells (Fig. 1D). Immunohistochemistry revealed strong diffuse positivity for S-100, HMB-45, and Melan-A, consistent with the diagnostic profile of metastatic malignant melanoma. Synthesizing the clinical history and ancillary investigations, the final diagnosis was confirmed as tracheal metastasis from a primary OMM. Despite the widespread dissemination of melanoma, tracheal metastasis remains a clinical rarity. Following confirmation of the diagnosis, the patient was transferred to an oncology specialty hospital for comprehensive individualized treatment.

## 3. Discussion

OMM primarily originates from melanocytes, and its development may be associated with specific genetic mutations in melanocyte stem cells or induced by external stimuli causing genetic alterations in mature melanocytes.^[3]^ During embryonic development, melanocyte precursors migrate from the neural crest along distinct pathways, with the majority ultimately settling in the epidermis and dermis of the skin, while a minority distribute themselves in the mucosal tissues of organs such as the respiratory tract, gastrointestinal tract, or urogenital tract.^[5,6]^ Therefore, OMM is not particularly common in itself, and its formation of metastases within the trachea is even rarer, accounting for <5% of all tracheal metastases caused by extrathoracic malignancies.^[7]^ The mechanism of tracheal metastasis remains incompletely understood; it may involve hematogenous dissemination or invasion of the trachea from adjacent tissues, such as the lymph nodes and lung parenchyma.^[8,9]^ The prognosis of distant metastasis is extremely poor.^[10]^ The clinical manifestations of tracheal metastasis of OMM are often nonspecific, with the most common symptoms including cough, dyspnea, and hemoptysis. Asymptomatic cases may be detected incidentally on chest imaging studies.^[11,12]^ Diagnosis presents considerable challenges owing to the nonspecific nature of the symptoms and imaging findings, which closely resemble those of primary lung cancer. Bronchoscopic biopsy is required to obtain tissue specimens for subsequent histopathology and immunohistochemistry to distinguish between primary tumors and extrapulmonary metastatic cancer.^[13]^ Therefore, bronchoscopy combined with histopathology and immunohistochemistry is crucial for a definitive diagnosis, particularly in the presence of pigmented lesions. Histopathology aids in clarifying the nature of the lesion, whereas immunohistochemistry can determine the tissue of origin, assisting in differentiating OMM from other malignancies and cutaneous melanomas. OMM typically shows positive staining for S-100, vimentin, HMB-45, and Melan-A, and negative staining for cytokeratin and epithelial membrane antigen.^[5]^ The patient presented with nonspecific respiratory symptoms and a history of primary oral melanoma. Computed tomography revealed soft tissue dense shadows within the trachea, and bronchoscopy revealed a pigmented neoplasm. Histopathology revealed melanoma cells, and immunohistochemistry showed strong positivity for S-100, HMB-45, and Melan-A. Therefore, a definitive diagnosis of OMM with tracheal metastasis was established.

Therapeutic options for OMM include surgery, chemotherapy, radiotherapy, immunotherapy, and targeted therapy. Surgical excision of the tumor is considered the gold standard treatment for OMM.^[14]^ However, surgical resection is generally only indicated for localized lesions, whereas systemic treatment regimens, such as immune checkpoint inhibitors, including ipilimumab, nivolumab, and pembrolizumab, are recommended for metastatic cases,^[5]^ significantly improving the prognosis of advanced disease. Studies have demonstrated that hTERT editing using the CRISPR-dCas9-dnmt3a system can inhibit melanoma cell growth, offering a novel therapeutic approach for melanoma treatment.^[15]^ In recent years, interventional bronchoscopy has emerged as an effective palliative treatment modality that can alleviate airway obstruction, improve symptoms, and enhance patients’ quality of life. Techniques such as high-frequency electrocautery, argon plasma coagulation, and cryotherapy offer advantages of reduced complications, preservation of the lung parenchyma, procedural simplicity, and the feasibility of repetitive interventions. In this patient, progressive airway obstruction caused by tracheal metastasis from OMM could be fatal, and appropriate management could be life-saving. Following bronchoscopic high-frequency electrocautery snare resection, the patient’s dyspnea symptoms improved. Despite diverse therapeutic approaches, the overall prognosis for tracheal metastasis of OMM remains poor because tracheal involvement signifies advanced disease, with a median post-metastasis survival of merely 6 to 16 months.^[16]^ Consequently, early detection of metastasis through rigorous surveillance, including regular imaging studies and bronchoscopy, may improve the patient’s quality of life.

This case report has several limitations. First, bronchoscopic high-frequency electrocautery snare resection can only alleviate the symptoms of tracheal lumen lesions without inhibiting the growth of melanoma. Second, the single-case design restricted the generalizability of the findings. Third, the absence of long-term follow-up data hindered the assessment of disease progression and treatment efficacy. Future studies should expand the patient cohorts and extend the observation periods to validate these findings and provide more reliable clinical evidence.

Future research should focus on the molecular mechanisms underlying tracheal metastasis in OMM and explore predictive biomarkers for early diagnosis to provide novel diagnostic tools for clinical practice. Furthermore, the emergence of intelligent decision-making systems integrated with advanced technologies such as the Internet of Things, machine learning, and blockchain will play an increasingly vital role in disease diagnosis.^[17,18]^ The convergence of these technologies holds promise for further advances in the precise diagnosis and personalized treatment of tracheal metastases.

## 4. Conclusion

Tracheal metastasis is an extremely rare complication of OMM. This paper reports a case of primary OMM complicated by tracheal metastasis, in which airway obstruction was successfully resolved via high-frequency electrocautery snare resection. Although tracheal metastasis is uncommon, it can be life-threatening. Bronchoscopic intervention can provide timely relief from airway symptoms in patients with tracheobronchomalacia. Therefore, tracheal metastasis should be suspected in patients with OMM who present with nonspecific respiratory symptoms. Prompt imaging and bronchoscopic examinations are essential for improving the patient’s quality of life.

## Acknowledgments

We would like to thank Editage for English language editing.

## Author contributions

**Conceptualization:** Yun Liu, Cuixia Bian.

**Data curation:** Shanshan Zhang.

**Methodology:** Wenwen Guo, Cuixia Bian.

**Supervision:** Cuixia Bian.

**Writing – original draft:** Yun Liu.

**Writing – review & editing:** Yun Liu, Cuixia Bian.
